# Accuracy of telepsychiatric assessment of new routine outpatient referrals

**DOI:** 10.1186/1471-244X-7-55

**Published:** 2007-10-05

**Authors:** Surendra P Singh, Dinesh Arya, Trish Peters

**Affiliations:** 1 Wolverhampton City Primary Care Trust, Mental Health Directorate, Steps to Health, Showell Circus, Low Hill, Wolverhampton WV10 9TH; University of Wolverhampton, UK; 2 Hawkes Bay District Health Board, Hawkes Bay Hospital, Omahu Road, Private Bag 9014, Hastings, New Zealand; 3 Mental Health Unit, Suite 1, Napier Health Centre, Hawkes Bay District Health Board, 76 Wellselsey Road, PO Box 447, Napier, New Zealand

## Abstract

**Background:**

Studies on the feasibility of telepsychiatry tend to concentrate only on a subset of clinical parameters. In contrast, this study utilises data from a comprehensive assessment. The main objective of this study is to compare the accuracy of findings from telepsychiatry with those from face to face interviews.

**Method:**

This is a primary, cross-sectional, single-cluster, balanced crossover, blind study involving new routine psychiatric referrals. Thirty-seven out of forty cases fulfilling the selection criteria went through a complete set of independent face to face and video assessments by the researchers who were blind to each other's findings.

**Results:**

The accuracy ratio of the pooled results for DSM-IV diagnoses, risk assessment, non-drug and drug interventions were all above 0.76, and the combined overall accuracy ratio was 0.81. There were substantial intermethod agreements for Cohen's kappa on all the major components of evaluation except on the Risk Assessment Scale where there was only weak agreement.

**Conclusion:**

Telepsychiatric assessment is a dependable method of assessment with a high degree of accuracy and substantial overall intermethod agreement when compared with standard face to face interview for new routine outpatient psychiatric referrals.

## Background

Verbal information and visual cues are major and primary ingredients of psychiatric assessment. The sounds and images transmitted through video-conferencing are equivalent to these two parameters respectively. Other factors such as empathy and rapport are also crucial and their influence on the outcome of assessment is well understood but not well quantified. The assumption that video-conferencing would provide results equivalent to those from face-to-face psychiatric interview is related to these corollaries and requires testing and quantification. Trust and confidence in using this technology can be greatly enhanced if this assumption is proved true. In view of rapid developments in hardware, wireless technology and data-transmission, psychiatric intervention through video-conferencing (telepsychiatry) can be an effective mode of service delivery, especially for remotely located population clusters.

Meeting mental health needs for remotely and sparsely populated communities has been a challenge to service providers due to various factors including resource constrains and difficulty in recruitment of mental health staff. Attempts have been made to address these concerns through the use of new and emerging technologies. When such new methods are used for clinical assessment, there is bound to be inherent uncertainties as to whether these new methods are as reliable, sensitive and accurate as existing methods of clinical assessment.

Although several studies [1-4] can be found on pilot projects and the feasibility of telepsychiatry, it seems that to date none have attempted to test a predetermined hypothesis for a complete set of clinical parameters in adult psychiatry. Earlier reviews [5-7] were complemented by evaluation of psychiatric assessment using the telephony system [8], video recording [9] and then video-conferencing [10,11]. A recent study [12] demonstrated usefulness of telepsychiatry as a valuable clinical and research tool. However, most of these studies focussed narrowly on the diagnostic aspect of psychiatric assessment. Other studies have attempted to deal with psychopathology [13,14], cost and feasibility [15,16], user satisfaction [3,17], acceptability [18] and psychological intervention [19,20]. The authors of this study failed to identify studies of reasonable quality on complete and comprehensive assessments of new psychiatric referrals in a general adult outpatient clinic.

This study attempts to detect the level of intermethod agreement between telepsychiatric assessment and face-to-face interview for routine new outpatient referrals to the general adult psychiatric unit. It is anticipated that there is a high level of agreement between conclusions drawn from psychiatric interviews through video-conferencing (V) and the standard method of face-to-face psychiatric assessment (S) for diagnosis, risk assessment and clinical intervention. This study aims to test this assumption.

## Methods

### Setting and participants

The study was conducted at Hawkes Bay Health Care, which provides a National Health Service to the Hawkes Bay and Chatham Island areas of New Zealand (NZ). The study was approved by the Hawkes Bay Ethics Committee, New Zealand. The sample consisted of consecutive new adult psychiatric referrals to the Napier Community Mental Health Team (NCMHT). They belonged to the 19 to 65 age group, were not under care of the NCMHT and had not received care for any mental health issue from this unit for a period of at least 6 months at the time of referral. Cases requiring urgent assessment or home visit were excluded.

In clinical practice, the outcome of the standard method of face-to-face assessment (S) is always supposed to be accurate. Accordingly, using method S as the gold standard, the results from V can be classified as 'accurate' if the outcome is identical to that from S for a given attribute, or as 'inaccurate' if there is disagreement between methods S and V. For the purpose of this study, the accuracy ratio (AR) is defined as the risk ratio (RR) between the accurate outcomes of video-assessment (V) and the results from face-to-face assessment (S). Assuming an AR of 0.95 or above for face-to-face assessment and results of video-assessment at a significance level of 0.05 and a power of 0.8, a sample size of 34 for each method would suffice to detect a difference of 15% or more between these two methods of assessment [21]. Accordingly, a sample of 40 participants based on single stage cluster sampling was considered to be adequate for this two-way, within subjects, crossed balanced design. The data derived from this study was also used for calculation of Cohen's kappa (CK) and its bootstrap confidence interval.

From the 40 consecutive new psychiatric referrals fulfilling above criteria, two cases declined to participate and one case could not be located. A written informed consent was obtained from all remaining 37 cases and they all went through their complete intended assessments. The referral period extended from 26 February 2001 to 15 May 2001 and the assessments were completed between 23 March 2001 and 17 May 2001.

### Assessment procedure

**Figure 1:**
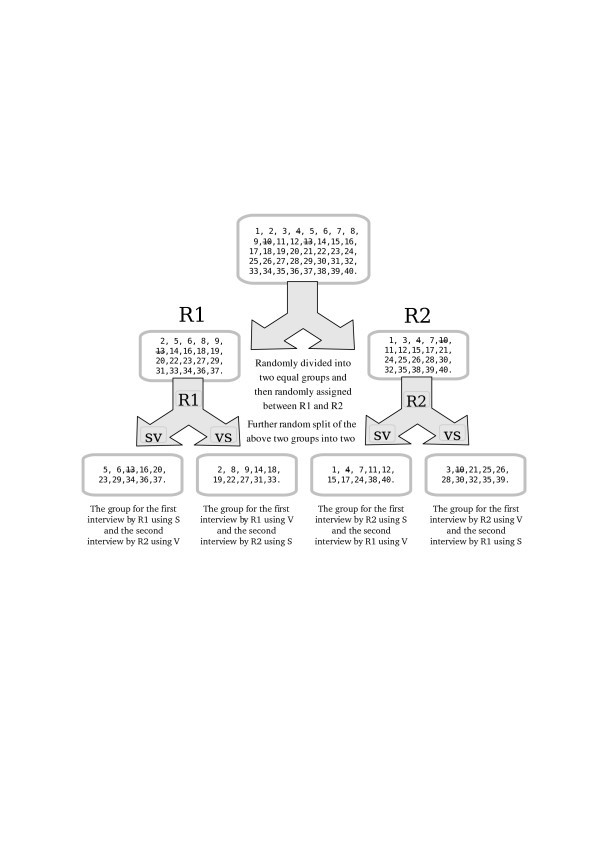
**The sample randomisation**. The numbers in the boxes are the serial numbers of the sample cases. Those crossed are drop-outs.

The assessment order for each method and for each psychiatrist was predetermined using a method of random allocation. The whole list was randomly divided into the two sub-lists, then participants were randomly allocated to have their first assessment either by researcher R1 or R2. The randomly selected half of the cases of each researcher (R1 and R2) had their first assessment by method S and the remaining half had their first assessment by method V. The second assessment (S or V as appropriate) of each individual case was subsequently completed by the other researcher (R1 or R2). The details of the randomisations and assessment procedures have been displayed in the Figure 1.

None of the researchers had prior experience of conducting formal telepsychiatric interviews for clinical care. Prior to initiating the research assessments, the researchers spent one session to familiarise with the equipment, and two sessions practising on known cases to evaluate and compare their findings in order to enhance their interrater agreement.

All face-to-face interviews were conducted at Hastings, while video assessment was carried out from Wairoa, 140 km away. All participants underwent both methods of assessment; each participant having one assessment on video by one psychiatrist and one face-to-face interview by the other psychiatrist. The interviewers utilised their own usual practice of clinical interview to resemble a standard outpatient setting.

The main confounding variables likely to influence the level of agreement are; bias between the researchers doing the assessments, duration of interview, use of interpreter, order-effect (effect on the second interview due to practice or residual memory from the first interview) and the time interval between the two methods of interview. The influence of such biases was minimised by adopting a crossover design and assigning an equal number of cases to each of the interviewing psychiatrists, to each interview methods, and to each order of assessments (S followed by V and V followed by S). The researchers were not aware of each others findings while assessing an individual participant. Both assessments for each participant were completed on the same date and each assessment lasted up to 60 minutes. If an interpreter was involved, he/she had to attend both sessions for that given case.

Video-Conferencing Units were available at Wairoa and Hastings. Both centres were equipped with a PictureTel Venue 2000 model 50 with 29 inch colour TV and were linked with a 384 KB (128 KB × 3) bandwidth ISDN line. Scanning and zooming of each of these video-conferencing units could be remotely controlled by the interviewer or by the interviewee. The Picture-in-picture (PIP) facility was not used on the interviewee side to prevent unnecessary distraction during the interview.

### Diagnostic tools, scales and data

Diagnoses on the DSM-IV axes were based on the method described in the Decision Trees for Differential Diagnosis of the Handbook [22] with assistance from the manual when required. A Risk Assessment Schedule (RAS) was adopted from the guidelines for assessment of risk factors identified by the NZ Ministry of Health [23]. This scale has not been tested for its reliability and validity and is included in the appendix for information [Appendix-I]. A List of Psychiatric Intervention (LIPI, Appendix-II) was developed to record options of admission/discharge/follow up, investigations, psychological intervention and community support. Primarily, this is a list of clinical decisions to select if applicable. The details of any pharmacological intervention were also recorded in a structured format.

The full diagnostic code for the DSM-IV-Axis-1, the presence or absence of diagnoses on Axis2 and Axis-3, the applicability or non-applicability of DSM-IV-Axis-4 questions and the score for DSM-IV-Axis-5 Global Assessment of Functioning (GAF) was recorded for each assessment. To confer uniformity, the numerical score of GAF was changed into ordinal type ranging from lower to high categories (A to E) based on a class interval method fulfilling the transformation criteria [24]. The RAS original scoring options of 'NIL' and 'LOW' were merged to 'low', and 'HIGH' and 'VERY HIGH' to 'serious'. This produced three distinct 'low', 'medium' and 'serious' risk categories for the purpose of statistical analysis. Possible responses for items of LIPI scale were dichotomous in nature excepting drug-related outcomes. Clinical decisions for investigations, psychological intervention and community support were summarised on a group wise basis. All medications were classified into nine types for eleven indications, resulting in five drug related initiatives.

Minor adjustments were made to present the data table in an n-by-n format as a pre-requisite for kappa calculation. One DSM-IV Axis-1 diagnosis of disorganised schizophrenia (V, 295.10) was changed to paranoid schizophrenia (295.30) giving a concordant entry; one case of cyclothymic disorder, (S, 301.13) was changed to bipolar disorder (296.56) giving a discordant entry; and one case of factitious disorder (V, 300.16) was changed to somatoform disorder (300.81) giving a concordant pair.

If the number of total diagnoses for a given case differed between methods of assessment, a category of 'NIL' was introduced to reflect the lack of identification of an equivalent diagnosis by the corresponding method. This led to the introduction of 2 concordant pairs and one discordant pair by the first method of adjustment and 3 discordant pairs arising from 'NIL' categories from the second method of adjustment. The resulting preponderance of discordant pairs over concordant pairs is likely to influence the interpretation against the research hypothesis, rather than in favour of it.

### Statistical evaluation

The test statistics of AR, Risk Difference (RD) and CK were calculated and summarised in accordance with methods described in the standard texts [24-26]. For the purpose of comparisons using AR and RD, an assumption is made that all outcomes from face-to-face assessment are 100% accurate. While using the asymptotic method of computation, some of the upper confidence interval of CK may exceed the permitted value of 1. Techniques like Bias Corrected Accelerated Bootstrap Confidence Interval (BCaCI) [27] and exact p estimate [28] have been advocated to resolve this paradox. Accordingly, this study applied non-parametric BCaCI methodology using 50,000 bootstrap samples with replacement.

The re-sampling was performed in a manner that retained the structural consistency of each subgroup. The techniques of bootstrapping and re-sampling are well established statistical methods and yet are little known in medical literature. The required software codes were developed for these models by the principal author (SPS) using R (version 2.4.1) [29] and were tested against other packages (SPSS, SAS and S-Plus) before data analysis. R is an open source statistical language software from the R Foundation of Statistical Computing, Vienna (ISBN 3-900051-07-0, 2006).

## Results

Of 37 participants, 20 were female and 17 male. Ethnically, there were 27 participants of European descent, 8 were of Maori origin and 2 were from other groups. Their ages ranged between 19.21 and 63.29 years; with an average age of 35.40 years and a standard deviation of 12.46 years.

The presence of statistical significance for the results of ARs in the Table 1 is based on two sided p value of 0.05 or less. The primary data from rows 1 to 30 in Table 1 and from rows 1 to 28 in Table 2 have been re-used to summarise in the remaining rows of their respective tables. This has invariably lead to multiple comparisons and interpretation of the results should reflect this limitation.

**Table 1:** Comparison of results of telepsychiatric assessments and face-to-face interviews

		**Sample**			**Accuracy Ratio Statistics**				**Risk Difference Statistics**			
**Primary attributes and accuracy**		**A**	**I**	**Size**	**AR**	**LCI**	**UCI**	**p**	**RD**	**LCI**	**UCI**	**p**
01	DSM-IV Axis 1	49	5	54	0.91	0.83	0.99	0.025	-0.09	-0.17	-0.02	0.019
02	DSM-IV Axis 2	34	3	37	0.92	0.84	1.01	0.083	-0.08	-0.17	0.01	0.071
03	DSM-IV Axis 3	36	2	38	0.95	0.88	1.02	0.157	-0.05	-0.12	0.02	0.146
04	DSM-IV Axis 4 Q1	31	6	37	0.84	0.73	0.97	0.014	-0.16	-0.28	-0.04	0.007
05	DSM-IV Axis 4 Q2	31	6	37	0.84	0.73	0.97	0.014	-0.16	-0.28	-0.04	0.007
06	DSM-IV Axis 4 Q3	37	0	37	1.00	1.00	1.00	NA	0.00	0.00	0.00	NA
07	DSM-IV Axis 4 Q4	36	1	37	0.97	0.92	1.03	0.317	-0.03	-0.08	0.03	0.311
08	DSM-IV Axis 4 Q5	37	0	37	1.00	1.00	1.00	NA	0.00	0.00	0.00	NA
09	DSM-IV Axis 4 Q6	32	5	37	0.86	0.76	0.98	0.025	-0.14	-0.25	-0.02	0.016
10	DSM-IV Axis 4 Q7	37	0	37	1.00	1.00	1.00	NA	0.00	0.00	0.00	NA
11	DSM-IV Axis 4 Q8	34	3	37	0.92	0.84	1.01	0.083	-0.08	-0.17	0.01	0.071
12	DSM-IV Axis 4 Q9	1	36	37	0.03	0.00	0.19	0.000	-0.97	-1.03	-0.92	0.000
13	DSM-IV Axis 5	26	11	37	0.70	0.57	0.87	0.001	-0.30	-0.44	-0.15	0.000
14	Risk to Self Q1	27	10	37	0.73	0.60	0.89	0.002	-0.27	-0.41	-0.13	0.000
15	Risk to Self Q2	29	8	37	0.78	0.66	0.93	0.005	-0.22	-0.35	-0.08	0.001
16	Risk to Self Q3	28	9	37	0.76	0.63	0.91	0.003	-0.24	-0.38	-0.10	0.001
17	Risk to Self Q4	28	9	37	0.76	0.63	0.91	0.003	-0.24	-0.38	-0.10	0.001
18	Risk to Others Q1	28	9	37	0.76	0.63	0.91	0.003	-0.24	-0.38	-0.10	0.001
19	Risk to Others Q2	35	2	37	0.95	0.88	1.02	0.157	-0.05	-0.13	0.02	0.146
20	Risk to Others Q3	33	4	37	0.89	0.80	1.00	0.046	-0.11	-0.21	-0.01	0.034
21	Risk to Others Q4	32	5	37	0.86	0.76	0.98	0.025	-0.14	-0.25	-0.02	0.016
22	Risk to Others Q5	33	4	37	0.89	0.80	1.00	0.046	-0.11	-0.21	-0.01	0.034
23	Risk to Others Q6	29	8	37	0.78	0.66	0.93	0.005	-0.22	-0.35	-0.08	0.001
24	Admit-Discharge-Follow-up	34	3	37	0.92	0.84	1.01	0.083	-0.08	-0.17	0.01	0.071
25	Investigations	25	12	37	0.68	0.54	0.84	0.001	-0.32	-0.48	-0.17	0.000
26	Psychological Input	26	11	37	0.70	0.57	0.87	0.001	-0.30	-0.44	-0.15	0.000
27	Community Support	29	8	37	0.78	0.66	0.93	0.005	-0.22	-0.35	-0.08	0.001
28	Drug Type	64	11	75	0.85	0.78	0.94	0.001	-0.15	-0.23	-0.07	0.000
29	Drug Action	58	17	75	0.77	0.68	0.87	0.000	-0.23	-0.32	-0.13	0.000
30	Drug Indication	49	26	75	0.65	0.55	0.77	0.000	-0.35	-0.45	-0.24	0.000

**Main Attributes**												

31	DSM-IV Axis 1 (1)	49	5	54	0.91	0.83	0.99	0.025	-0.09	-0.17	-0.02	0.019
32	DSM-IV Axis 2 (2)	34	3	37	0.92	0.84	1.01	0.083	-0.08	-0.17	0.01	0.071
33	DSM-IV Axis 3 (3)	36	2	38	0.95	0.88	1.02	0.157	-0.05	-0.12	0.02	0.146
34	DSM-IV Axis 4 (4:12)	276	57	333	0.83	0.79	0.87	0.000	-0.17	-0.21	-0.13	0.000
35	DSM-IV Axis 5 (13)	26	11	37	0.70	0.57	0.87	0.001	-0.30	-0.44	-0.15	0.000
36	Risk to Self (14:17)	112	36	148	0.76	0.69	0.83	0.000	-0.24	-0.31	-0.17	0.000
37	Risk to Others (18:23)	190	32	222	0.86	0.81	0.90	0.000	-0.14	-0.19	-0.10	0.000
38	Admit-Discharge-Follow-up (24)	34	3	37	0.92	0.84	1.01	0.083	-0.08	-0.17	0.01	0.071
39	Investigations (25)	25	12	37	0.68	0.54	0.84	0.001	-0.32	-0.48	-0.17	0.000
40	Psychological Input (26)	26	11	37	0.70	0.57	0.87	0.001	-0.30	-0.44	-0.15	0.000
41	Community Support (27)	29	8	37	0.78	0.66	0.93	0.005	-0.22	-0.35	-0.08	0.001
42	Drug Type (28)	64	11	75	0.85	0.78	0.94	0.001	-0.15	-0.23	-0.07	0.000
43	Drug Action (29)	58	17	75	0.77	0.68	0.87	0.000	-0.23	-0.32	-0.13	0.000
44	Drug Indication (30)	49	26	75	0.65	0.55	0.77	0.000	-0.35	-0.45	-0.24	0.000

**Major Attributes**												

45	DSM Diagnosis (1:13)	421	78	499	0.84	0.81	0.88	0.000	-0.16	-0.19	-0.12	0.000
46	Risks (14:23)	302	68	370	0.82	0.78	0.86	0.000	-0.18	-0.22	-0.14	0.000
47	Non-Drug Intervention (24:27)	114	34	148	0.77	0.71	0.84	0.000	-0.23	-0.30	-0.16	0.000
48	Drug Intervention (28:30)	171	54	225	0.76	0.71	0.82	0.000	-0.24	-0.30	-0.18	0.000

**Overall**												

**49**	**Overall Result (1:30)**	**1008**	**234**	**1242**	**0.81**	**0.79**	**0.83**	**0.000**	**-0.19**	**-0.21**	**-0.17**	**0.000**

A: Accurate outcome, I: Inaccurate Outcome, AR: Accuracy Ratio (Risk Ratio), LCI: Lower 95% Confidence Interval, UCI: Upper 95% Confidence Interval, RD: Risk Difference when the sample statistics compared with Accuracy (Risk) of 1 for the face-to-face interview. All approximated p values are more than zero.

Numbers in brackets represent index number of primary attributes serialised in the previous rows.

Drug Types- Atypical Antipsychotic, Mood Stabilizer, Tricyclic Antidepressant, Newer Antidepressant, Other Antidepressant, Benzodiazepine Group, Non-Benzodiazepine Anxolytic, Anti-Parkinsonian, and 'No Medication'.

Drug related actions were grouped into 'New Prescription', 'Continuation of previously prescribed medication with no change', and 'Adjustment of previously prescribed medication'.

Drug Indications were categorised for Positive Psychotic Symptoms, Manic symptoms, Bipolar Disorder, Depression, Anxiety and Associated Symptoms, Drugs for Side Effects (e.g. Anti-parkinsonian), Hypnotics, and Use of Drugs' sedative effect for anxiety control and sleep.

**Table 2:** Cohen's Kappa results of intermethod and interviewers assessments

				**Standard vs Video**			**Interviewers**		
**SN**	**Primary Attributes**	**Size**	**Group**	**CK**	**LCI**	**UCI**	**CK**	**LCI**	**UCI**
1	DSM-IV Axis 1	54	26	**0.90**	0.28	1.00	**0.90**	0.33	1.00
2	DSM-IV Axis 2	37	2	0.62	-0.04	0.87	**0.63**	0.21	0.87
3	DSM-IV Axis 3	38	2	**0.84**	0.49	0.93	**0.84**	0.49	0.93
4	DSM-IV Axis 4 Q1	37	2	**0.57**	0.25	0.82	**0.57**	0.22	0.83
5	DSM-IV Axis 4 Q2	37	2	0.17	-0.11	0.72	0.18	-0.09	0.68
6	DSM-IV Axis 4 Q3	37	2	**0.85**	0.66	0.91	**0.85**	0.66	0.91
7	DSM-IV Axis 4 Q4	37	2	**0.87**	0.65	0.94	**0.87**	0.65	0.94
8	DSM-IV Axis 4 Q5	37	2	**0.66**	NA	NA	**0.66**	NA	NA
9	DSM-IV Axis 4 Q6	37	2	**0.55**	0.18	0.84	**0.54**	0.13	0.84
10	DSM-IV Axis 4 Q8	37	2	0.36	-0.07	0.79	0.37	-0.08	0.54
11	DSM-IV Axis 5	37	5	**0.89**	0.81	0.94	**0.89**	0.81	0.94
12	Risk to self Q1	37	3	**0.66**	0.39	0.86	**0.65**	0.39	0.86
13	Risk to self Q2	37	3	**0.68**	0.34	0.86	**0.68**	0.37	0.86
14	Risk to self Q3	37	3	0.55	-0.11	0.73	**0.55**	0.22	0.80
15	Risk to self Q4	37	3	0.45	-0.11	0.68	**0.45**	0.12	0.79
16	Risk to others Q1	37	3	**0.62**	0.27	0.86	**0.62**	0.27	0.86
17	Risk to others Q2	37	3	**0.82**	0.30	1.00	**0.82**	0.37	1.00
18	Risk to others Q3	37	2	-0.04	-0.10	-0.03	-0.04	-0.10	-0.03
19	Risk to others Q4	37	3	0.55	-0.11	0.78	0.55	-0.06	0.78
20	Risk to others Q5	37	2	0.44	-0.07	0.84	0.44	-0.06	0.84
21	Risk to others Q6	37	3	0.41	-0.13	0.66	0.41	-0.12	0.66
22	Admit-Discharge-Follow up	37	3	**0.76**	0.37	0.93	**0.76**	0.37	0.93
23	Investigations	37	2	0.29	-0.03	0.59	0.30	-0.02	0.59
24	Psychological Input	37	2	0.16	-0.17	0.53	0.18	-0.12	0.54
25	Community Support	37	2	**0.55**	0.22	0.78	**0.56**	0.25	0.78
26	Drug Type	75	9	**0.83**	0.61	0.90	**0.82**	0.52	0.90
27	Drug Action	75	5	**0.67**	0.52	0.79	**0.66**	0.52	0.79
28	Drug Indication	75	11	**0.59**	0.35	0.74	**0.59**	0.35	0.76

**Main Attributes**									

29	DSM-IV Axis 1 (1)	54	26	**0.90**	0.28	1.00	**0.90**	0.33	1.00
30	DSM-IV Axis 2 (2)	37	2	0.62	-0.04	0.87	**0.63**	0.21	0.87
31	DSM-IV Axis 3 (3)	38	2	**0.84**	0.49	0.93	**0.84**	0.49	0.93
32	DSM-IV Axis 4 (4:10)	259	14	**0.65**	0.52	0.78	**0.65**	0.52	0.78
33	DSM-IV Axis 5 (11)	37	5	**0.89**	0.81	0.94	**0.89**	0.81	0.94
34	Risk to self (12:15)	148	12	**0.61**	0.48	0.75	**0.61**	0.48	0.75
35	Risk to Others (16:21)	222	16	0.05	-0.01	0.11	0.05	-0.01	0.11
36	Admit-Discharge-Follow up (22)	37	3	**0.76**	0.37	0.93	**0.76**	0.37	0.93
37	Investigations (23)	37	2	0.29	-0.03	0.59	0.30	-0.02	0.59
38	Psychological Input (24)	37	2	0.16	-0.17	0.53	0.18	-0.12	0.54
39	Community Support (25)	37	2	**0.55**	0.22	0.78	**0.56**	0.25	0.78
40	Drug Type (26)	75	9	**0.83**	0.61	0.90	**0.82**	0.52	0.90
41	Drug Action (27)	75	5	**0.67**	0.52	0.79	**0.66**	0.52	0.79
42	Drug Indication (28)	75	11	**0.59**	0.35	0.74	**0.59**	0.35	0.76

**Major Attributes**									

43	DSM-IV (1:11)	425	49	**0.86**	0.81	0.90	**0.86**	0.81	0.90
44	Risks (12:21)	370	28	**0.14**	0.08	0.19	**0.14**	0.09	0.19
45	Non-Drug Intervention (22:25)	148	9	**0.49**	0.35	0.64	**0.49**	0.35	0.64
46	Drug Intervention (26:28)	225	25	**0.72**	0.66	0.79	**0.72**	0.66	0.79

**Overall**									

47	Overall Result (1:28)	1168	111	**0.60**	0.57	0.63	**0.60**	0.57	0.63

The column 'Size' constitutes the number of total sample points used for assessments.

The column 'Group' stands for the number of total categories of results e.g. all diagnoses with Paranoid Schizophrenia will make one group.

CK: Cohen's Kappa values derived from 'n by n' table made from the Groups. The significant values (Lower CI more than 0) are printed in bold. The summary CK results in the rows from 29 to 47 are based on "Inverse Variance method" using CKs and variances from previous rows displayed in the brackets for the respective assessments.

95% Lower & Upper Confidence Intervals (LCI and UCI) from the rows 1 to 28 are derived from non-parametric Bias Corrected Accelerated (BCa) variance from 50,000 bootstrap samples with replacement. The summary LCIs and UCIs in the rows from 29 to 47 are based on "Inverse Variance method" using CKs and original variances from previous rows are displayed in the brackets for the respective assessments.

Weighted Kappa using "square error weights" were computed for ordinal observations where applicable.

The definitions for Drug Type, Drug Action and Drug Indication are same as in the Table 1.

The ARs (Table 1) with nil variance (rows 6, 8, 10) were excluded from comparison due to the constant nature of observation data. The results with upper 95% confidence interval of AR>1 (rows 2, 3, 7, 11, 19, 24) were not treated as statistically significant due to the fact that the accuracy ratio cannot exceed a maximum value of 1. Using these criteria, all the remaining observations of the 'primary attributes' are both valid and statistically significant between 0.65 and 0.91 excepting one of the items (row 12) in Axis 4 of the DSM. The pooled ARs for the main attributes (rows 31, 34 to 37, and 39 to 44) and major attributes (rows 45 to 48) are from 0.65 to 0.91 and from 0.76 to 0.84 respectively. The overall AR (row 49) for the combined assessments is 0.81.

The criteria described in the previous paragraph were also applied for RDs (Table 1). Accordingly, all the valid observations excluding DSM-IV Axis 4 Q9, range between -0.35 and -0.09 and are statistically significant. There is an overall accuracy difference of -0.19, with 95% confidence interval between -0.21 and -0.17. This result supports a hypothesis that overall outcome of telepsychiatric assessment is about 19% inferior to face-to-face interview.

Table 2 has observation data about agreements between the two methods of interview and between the interviewing psychiatrists. There is trend to classify CK values ≤ 0 as poor, those from 0.01 to 0.20 as slight, from 0.21 to 0.40 as fair, from 0.41 to 0.60 as moderate, from 0.61 to 0.80 as substantial and from 0.81 to 1 as perfect [30]. From a total of 27 valid primary attributes (rows 1 to 28), 16 have moderate to substantial statistically significant intermethod BCa Kappa values. Similarly, 10 of 14 main attributes (rows 29 to 42) have agreements at moderate to substantial level.

The Kappa scores for intermethod agreement of DSM Axes (rows 29 to 33) varied from substantial (0.65) to perfect (0.90) excepting axis 2, where the result was not statistically significant. The agreement was perfect (0.86) for combined DSM categories (row 43). The overall Kappa score for risk (row 44) was only slight, though it was substantial in respect of assessment of 'risk to self' (row 34). Agreement levels for investigations and psychological input (rows 23 and 24) were non-significant while that for Community Support (row 25) was moderate (0.55). There was moderate agreement (0.49) on non-drug intervention as a whole (row 45). Various components of Drug Treatment (rows 26 to 28) had agreement levels between high moderate (0.59) to perfect (0.83) with substantial rating (0.72) as a whole (row 46). Overall agreement between the telepsychiatric assessments and face-to-face interviews for the completed psychiatric assessments reached a downward approximated value of 0.60, hence it was substantial.

The interrater agreements between the two interviewing psychiatrists were very close and could not be differentiated from that of intermethod assessment. Their respective values do not differ more than 0.01, with a considerable degree of overlap between their confidence intervals. Accordingly, there is no significant difference between intermethod and interrater agreements and are equivalent to each other.

## Discussion

This study aims to establish whether conclusions drawn from telepsychiatric assessments are in agreement with those from the standard method of face-to-face assessment for new referrals in an outpatient clinic. Most new and old referrals to the Community Mental Health Teams in the UK and NZ are discussed in a multidisciplinary team setting. Application of DSM diagnostic criteria in NZ has gradually evolved into standard clinical practice and is becoming popular in the UK. The application of Decision Tree for diagnosis on Axis-1 of DSM-IV can be easily adopted without significant additional resources and training. The methodology adopted in this study emulates the real clinic situation; hence its findings are both applicable and relevant to day-to-day clinical practice.

The authors have taken all necessary precautions in dealing with anticipated results, some of which might be paradoxical or erroneous e.g. CK and AR exceeding a maximum permitted value of 1. Those results where AR>1 and with nil variance were excluded from further statistical interpretation. Though the number of studied cases was only 37, the numbers of sample points (as in 'Size' column of Table 1) for statistical calculation were large enough for meaningful interpretation. The issue of sample size in handling large number of diagnostic categories for CK was dealt with bootstrapping technique and non-parametric Bias Corrected Accelerated Bootstrap Confidence Interval. This approach enhances the statistical quality of the data analysis.

A previous study [5] evaluating twelve telepsychiatric and face-to-face assessments on multiple scales found a mean weighted kappa coefficient of 0.85. The interrater reliability for diagnosis between two psychiatrists in three different experimental conditions on 63 patients has been found to vary between 0.69 and 0.85 [6]. A review of telepsychiatry services in Australia concluded that this technology could be reliably used for treatment recommendations and diagnostic assessments [7]. A Canadian study involving child psychiatrists found that in 96% of cases, the diagnosis and treatment recommendations made via video-conferencing were identical to those made in face-to-face interviews [10]. Another study [8] using telecommunication and audiovisual technology found interrater diagnostic agreement of 0.70. Despite methodological differences, the results from the present study are consistent with the findings quoted above in this paragraph.

Interrater agreement among clinicians for video-taped face-to-face interviews has been noted to have a relatively lower CK value of 0.55 [9]. It is possible that the flexibility conferred by the ability to question the patient in real time in a face-to-face or video-conferencing interview is an advantage over videotape assessment and accounting for improved intermethod and interrater agreement.

In a field trial of DSM-III, the interrater reliability for face-to-face interviews for the major disorders varied between kappa values of 0.28 to 0.92 [31] and an another study on ICD-10 also yielded a fair to good kappa values for the four-character diagnostic codes [32]. In comparison, the outcome of telepsychiatric assessments in the current study is at least similar to the interrater reliability of face-to-face interviews from these large field trials.

None of the primary studies quoted above tested intermethod agreement for a complete set of clinical parameters. Their sample size and statistical methodology are also limiting factors for satisfactory conclusions. In contrast, the present study employs suitable statistical methods for comprehensive outpatient assessment for multi-axial DSM-IV diagnosis, risk assessment, investigations, and treatment. As compared to standard method of face-to-face interviews, telepsychiatric assessments in this study have a high accuracy ratio (AR 0.81) and a substantial intermethod agreement (CK 0.60).

Although the kappa value of intermethod agreement for risk assessment is low, it would be premature to ascribe this to telepsychiatric assessment itself. A study on videotaped interviews of 30 patients attending emergency psychiatry service revealed interrater correlation coefficients of 0.32 and 0.44 for risks to self and others respectively [33]. Another study conducted in a comparable setting also found similar results and came with the observation that in some circumstances the level of disagreement was high enough to warrant concern [34]. In a prospective study for risk assessment on 161 inmates of a high risk forensic unit [35] the agreement level among psychiatrists for face-to-face interviews in absence of operational criteria was very poor (CK -0.006). The same study reported that this agreement can be greatly enhanced (CK 0.742) by application of operational criteria. The findings in the present study for risk assessment are comparable to these results. In addition, the lower base level of risk in routine outpatient clinics in comparison to emergency and forensic psychiatric units is likely to cause further decrement in the kappa level.

There is a paucity of research-based knowledge concerning levels of agreement for risk factors. Most of the scales currently used for risk-assessments have yet to have their reliability, validity and predictability ascertained. To establish agreement levels in statistical terms for uncommon risk elements (suicides and homicides etc.) would require an enormous sample which may not be feasible. Lack of a valid and reliable tool for risk assessment may produce erroneous results while uncertainty over the time frame (short-term, immediate and long-term) for risk anticipation may lead to inconsistencies in reporting and recording. The sort-term serious risk will probably be dealt with in the emergency system rather than through routine outpatient referrals and the long-term risks are likely to have minimal influence on the decision making process while dealing with routine outpatient referrals.

The ability to reach an accurate DSM-IV-Axis-1 diagnosis through telepsychiatric assessment is perfect (CK 0.90) and accurate (AR 0.91). Arriving at a reasonable diagnostic impression is a pre-requisite of the medical recommendation for assessment or treatment under the Mental Health Act and this objective can be very well achieved through telepsychiatry. Another prerequisite under the act is to evaluate potential risks with input and information from various sources. Identification of risk related concerns direct from the interviewee constitutes only one component of this whole process. The referring agency usually indicates and expresses its concerns about risk elements and additional information are generally obtained on telephone from other sources such as clinicians and family members. With this in mind, low concordance on risk assessment may not necessarily be a limiting factor in the use of telepsychiatry for the purpose of Mental Health Act assessment.

## Conclusion

Telepsychiatry is a dependable mode of service delivery for diagnostic assessment and psychiatric intervention in routine new referrals. Its accuracy varies between 79% and 83% in comparison with face-to-face interview. There is also an overall substantial agreement between these two methods of psychiatric evaluation. Although there is potential for usage of telepsychiatry for the Mental Health Act assessment, this requires further research using more refined operational tools to enhance the low accuracy and agreement scores found in the present study. The accuracy of conclusions arrived at from telepsychiatric assessment is likely to improve in future with further advances in technology [36].

### Clinical implications

1. Allows telepsychiatric services to be made available to a geographically distant and inaccessible population where it is difficult and expensive to recruit mental health professionals.

2. Enhances confidence in use of telepsychiatry as an alternative mode of service delivery.

3. Increases scope of international research and collaboration in the practice of clinical psychiatry in different parts of the world.

### Limitations and solutions

1. Although the outcome of risk assessment was similar to other studies, the level of agreement for this parameter is significantly low. There is scope to overcome this deficit through usage of operational criteria [35]. On this subject, some of the scientifically unexplored topics such as tools for risk assessment, its reliability and predictive value require further research.

2. The study assumed that there is 100% concordance between clinical decisions amongst psychiatrists if they conduct face-to-face interviews. This is seldom the case. Further studies with an added component to detect overall interrater agreement for face-to-face assessment will help in eliminating the need for this hypothetical 100% concordance rate.

3. There is an inherent problem in determining the sample size for CK for an unknown number of categories that may be encountered during a prospective research. This requires application of alternative statistical approaches. The current study has attempted to address some of these concerns through usage of resampling method and bootstrap confidence intervals.

## List of Abbreviations used

APA: American Psychiatric Association

AR: Accuracy Ratio

BCaCI: Bias Corrected Accelerated Bootstrap Confidence Interval

CK: Cohen's Kappa

DSMIV: Diagnostic and Statistical Manual of Mental Disorders – 4^th ^edition

GAF: Global Assessment of Functioning

HBHB: Hawkes Bay Health Board, New Zealand

ICD: International Classification of Diseases

ISDN: Integrated Services Digital Network

KB: Kilo bits per second

LIPI: List of Psychiatric Intervention

LCI: Lower 95% Confidence Interval

NZ: New Zealand

NCMHT: Napier Community Mental Health Team

R: Open source statistical package similar to S-Plus [29]

R1: Researcher 1 – Surendra Singh

R2: Researcher 2 – Dinesh Arya

RAS: Risk Assessment Schedule

RD: Risk Ratio

RR: Relative Risk

S: Standard method of face to face psychiatric assessment

S-Plus: The statistics package built upon the S programming language

SAS: The Statistical Analysis System from SAS Institute

SPSS: The Statistical Package for the Social Sciences

SPS: Principal author S P Singh

UCI: Upper 95% Confidence Interval

UK: United Kingdom

V: Video-conferencing

## Competing interests

The author(s) declare that they have no competing interests.

## Authors' contributions

Surendra P Singh

Planning, design, resource arrangement, coordination, template design, clinical assessment, data collection, data entry, statistical consideration, data analysis, review, writing and submission.

Dinesh Arya

Consultation and suggestion; especially about risk assessment and elements of statistical consideration, clinical assessment and data collection.

Trish Peters

Randomisation, liaison, consenting and coordination with other researchers and participants.

All authors have read and approved the submitted manuscript.

## Appendix-I

Table 3

**Table 3:** 

**RAS (Risk Assessment Schedule)** Enter the RISK in terms of NIL, LOW, MEDIUM, HIGH & VERY HIGH Risk.					
**MENTAL STATE (RAS – 1)**	**N**	**L**	**M**	**H**	**V**

**Behaviour**					
• Dangerous or threatening actions	N	L	M	H	V
• Verbal/non-verbal risks	N	L	M	H	V
• Deliberate self harm	N	L	M	H	V
• Aggression	N	L	M	H	V

**Affect**					
• Arousal, anger, hostility, irritability, suspiciousness, fear	N	L	M	H	V
• Low mood or elevated mood	N	L	M	H	V

**Cognition**					
• Fantasies of deliberate self harm or harm to others	N	L	M	H	V
• Persecutory thoughts, delusions	N	L	M	H	V
• External control	N	L	M	H	V
• Confusion	N	L	M	H	V
• Preoccupation, obsession, jealousy	N	L	M	H	V
• Control over-ride	N	L	M	H	V
• Cultural beliefs	N	L	M	H	V

**Perceptions**					
• Command hallucinations	N	L	M	H	V
• Misidentification	N	L	M	H	V
• Matakite	N	L	M	H	V

**ENVIRONMENTAL/CURRENT FACTORS (RAS – 2)**	**N**	**L**	**M**	**H**	**V**

**Immediate stressors**					
• Substance use, intoxication or withdrawal	N	L	M	H	V
• Relationships	N	L	M	H	V
• Presence or absence of support	N	L	M	H	V
• Absence of treatment, non compliance	N	L	M	H	V
• Persecution or threats from others	N	L	M	H	V
• Arrest or criminal charges	N	L	M	H	V
• Loss including death of a peer	N	L	M	H	V
• Cultural transgression	N	L	M	H	V
• Financial stress	N	L	M	H	V

**Access**					
• To weapons, pills, victims	N	L	M	H	V

**Situation**					
• Referral from Prison, Police, Secure Unit	N	L	M	H	V

**Individual's attitude**					
• Co-operation	N	L	M	H	V
• Refusal to co-operate or fear of compulsory treatment	N	L	M	H	V

**HISTORICAL INFORMATION (RAS-3)**	**N**	**L**	**M**	**H**	**V**

**Illness and incidents**					
• Patterns of illness – Chronic active, Neurological disorder, H/O Head injury	N	L	M	H	V
• Psychiatric history – Serious mental illness, Multiple diagnoses, Treatment under MHA	N	L	M	H	V
• History of incidents (and context) – Repeated antisocial behavior	N	L	M	H	V
• Treatment and outcomes – Compliance and response of treatment given in past	N	L	M	H	V
• Features of past crises – Pathological intoxication, Episodic dyscontrol, Cruelty to animals, Blackouts	N	L	M	H	V
• Personal history – Criminal charges, Previous offenses, Forensic involvement	N	L	M	H	V

**Personality**					
• Usual coping style – Loner, Displaced rage reaction, self mutilism, Impulsivity, denial, Blaming others	N	L	M	H	V

**Family background**					
• Demographics – Single, Male, Poor, Low educational and vocational success	N	L	M	H	V
• Culture – Tolerance to antisocial behavior, Reluctance to disclose, Shame & Guilt	N	L	M	H	V
• Dynamics – H/O Violence, Contact of violent gangs, Intrafamilial violence	N	L	M	H	V

**OUTCOME OF RISK ASSESSMENT** (Use knowledge & Information gathered from RAS-1, 2 & 3)					

**RISK TO SELF**	**N**	**L**	**M**	**H**	**V**

1. Safety (including suicidal acts, deliberate self harm)	N	L	M	H	V
2. Health (incl. drug & alcohol abuse, physical & psychological harm)	N	L	M	H	V
3. Self neglect and vulnerability (incl. exploitation, sexual abuse, violence from others)	N	L	M	H	V
4. Quality of life (including dignity, social and financial status)	N	L	M	H	V

**RISK TO OTHERS**	**N**	**L**	**M**	**H**	**V**

1. Violence (including emotional, sexual and physical violence)					
2. Intimidation/threats	N	L	M	H	V
3. Neglect/abuse of Dependants	N	L	M	H	V
4. Stalking/harassment	N	L	M	H	V
5. Reckless behavior (including driving)Property damage (including arson)	N	L	M	H	V
6. Public nuisance	N	L	M	H	V

## Appendix-II

Table 4

**Table 4:** LIST OF PSYCHIATRIC INTERVENTION (LIPI)

**Admission/Discharge/Followup (LIPI-1):**
Admission
Discharge
Followup

**Investigations (LIPI-2):**
Haematological
Biochemical
Serum Level of Medication
ECG tests
EEG Examination
CT/NMRI/PET/Isotope/Ultrasound
IQ Assessment
Other biological test
Other Psychological Tests

**Psychological Intervention (LIPI-3):**
Investigation
Simple Explanations
Support & Advice
Self Help by Book
Self Help Group
Assertiveness Training
Anger Control
Domestic Skills Training
Budget Handling Training
Social Skill Training
Counselling
Marital Therapy
Divers ional Therapy
Relaxation Training
Paper bag Ventilation
Systemic Desensitisation
Biofeedback
Cognitive Behaviour Therapy
Psychotherapy
Family Therapy
Other

**Community Support (LIPI-4):**
**Intervention**
CPN visit to support patient
CPN visit to support family
Activity Therapy (Gym, sports etc)
Handling Bills and Finances
Helping in making Applications to other agencies
Outreach
Home Help
Meals on Wheels
Art & Work therapy (Art, wood, gardening)
Day Hospital for socialisation

**LIPI-5**: Information related to Drug Type, Action and Indication scored in different table

## Pre-publication history

The pre-publication history for this paper can be accessed here:


